# 

**Published:** 1921-02-12

**Authors:** 


### Matrons' and Sisters' Appointments.

UNION HOSPITAL, STOKE-ON-TRENT.—Miss H. M. Pountney has been appointed night sister. She was trained at Chesterfield Union Hospital and at the Jessop Hospital, Sheffield. After her training she engaged in private nursing at Clevedon, and for a time was temporary matron of the Cottage Hospital, Clevedon.

GENERAL HOSPITAL, BIRMINGHAM.—Miss P. Gray Gibson has been appointed night sister. She was trained at the Royal Victoria Infirmary, Newcastle-on-Tyne, and then joined the private staff of Preston Royal Infirmary. She subsequently filled the posts of charge sister at the Central Nursing Home, Nottingham, ward and theatre sister at Bethnal Green Infirmary, and charge sister at Romford Military Hospital. She holds the certificate of the Central Midwives Board.

ROYAL MIDLAND COUNTIES HOME FOR INCURABLES, LEAMINGTON.—Miss A. M. Oldham has been appointed assistant matron. She was trained at Smallwood Hospital, Redditch, where she was later staff nurse, which post she subsequently filled at the Llandrindod Wells Hospital.

CENTRAL HOME INFIRMARY, PONTYPRIDD.—Miss Mary Elizabeth Price has been appointed sister. She was trained at the Merthyr Tydfil Infirmary, and has been staff nurse at the Medway Infirmary, Chatham, later taking midwifery training at the Maternity Hospital, Loveday Street, Birmingham. She has since seen military service under the Q.A.I.M.N.S.

### Personalia.

SIR HARRIS SPENCER, K.B.E., has been elected honorary treasurer to the West Bromwich and District Hospital. Dr. H. Black has been appointed honorary radiographer.

MISS GERALDINE MATHEWS, R.R.C., O.B.E., late matron of the National Children's Hospital, Dublin, was married at St. Anne's Parish Church, Dublin, on February 1, to Lieut.-Col. Sir Lambert H. Ormsby, M.D., F.R.C.S., of Merrion Square, Dublin. The bride is well known in Dublin, and rendered distinguished service in aiding wounded persons under fire. Her personal efforts and conspicuous bravery were the means of saving three lives. She also rendered valuable aid to sick and wounded officers and soldiers during the war. Lady Ormsby is the only daughter of the late William Mathews, Esq., of Hyères, Var, France.

